# Synovial fluid dual‐biomarker algorithm accurately differentiates osteoarthritis from inflammatory arthritis

**DOI:** 10.1002/jor.26005

**Published:** 2024-12-18

**Authors:** Daniel Keter, Van Thai‐Paquette, John Miamidian, Simmi Gulati, Krista Toler

**Affiliations:** ^1^ Research and Development CD Diagnostics, A Division of Zimmer Biomet Claymont Delaware USA; ^2^ CD Laboratories Towson Maryland USA

**Keywords:** cartilage oligomeric matrix protein (COMP), diagnosis, inflammatory arthritis, Interleukin‐8 (IL‐8), osteoarthritis (OA)

## Abstract

Osteoarthritis (OA) prevalence increases as the population ages. Diagnosing osteoarthritis often occurs in the late stages when cartilage degradation is severe, making it difficult to distinguish from other types of arthritis. Accurate differentiation of primary osteoarthritis from other arthritic conditions is crucial for effective treatment planning. A new diagnostic test has been developed that uses a dual‐biomarker algorithm to inform osteoarthritis diagnosis. Synovial fluid from patients with confirmed primary osteoarthritis showed elevated levels of cartilage oligomeric matrix protein. However, this biomarker alone could not distinguish primary osteoarthritis from other inflammatory conditions that also cause cartilage deterioration. Therefore, a combinatorial algorithm using cartilage oligomeric matrix protein and Interleukin‐8 concentrations was developed to differentiate primary osteoarthritis from inflammatory arthritis. Clinical decision limits for cartilage oligomeric matrix protein concentration and the cartilage oligomeric matrix protein to Interleukin‐8 ratio were established and validated using 171 human knee synovial fluid specimens. The osteoarthritis algorithm demonstrated clinical sensitivity and specificity of 87.0% and 88.9%, respectively. This is the first report of a biomarker test that can differentiate primary osteoarthritis from inflammatory arthritis with a high degree of accuracy.

## Clinical Significance

This study introduces a novel dual‐biomarker algorithm utilizing synovial fluid analysis, offering a significant advancement in accurately diagnosing osteoarthritis, particularly in distinguishing it from inflammatory arthritis. This approach holds promise for improving clinical decision‐making and patient outcomes, addressing a critical unmet need in arthritis diagnosis.

## Graphical abstract

This study addresses an unmet need for objective diagnosis of osteoarthritis to improve clinical decision‐making and patient outcomes. Testing of human knee synovial fluid specimens that represented osteoarthritis, rheumatoid arthritis, crystalline arthritis, and native septic arthritis was carried out using internally and externally validated synovial fluid assays. A combinatorial algorithm utilizing cartilage oligomeric matrix protein, and Interleukin‐8 concentrations was developed that differentiated primary osteoarthritis from inflammatory arthritis. The clinical sensitivity and specificity of the osteoarthritis algorithm were 87.0% and 88.9%, respectively, with an overall accuracy of 88.3%. (A) Discovery data demonstrating the utility of COMP and IL‐8 as a dual biomarker algorithm in differentiating osteoarthritis from other arthropathies. (B) Algorithm decision outline to determine whether samples were positive for primary OA.

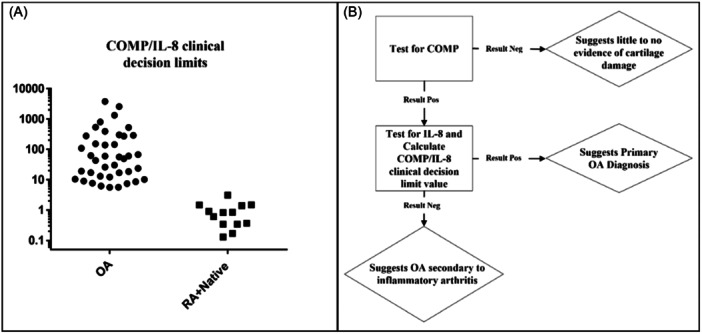

## 1 INTRODUCTION

Osteoarthritis (OA) is the most common cause of musculoskeletal pain and chronic disability of synovial joints worldwide.^1^ In the United States, OA occurs in 10−15% of people aged 60 and older, and its incidence is expected to increase due to the aging population and other personal risk factors, such as obesity. There have been observed differences in arthritis and associated conditions between populations of different age and gender.^2^, ^3^ The risk of decreased mobility attributable to OA is greater than any other medical condition in people aged 65 and older.^4^, ^5^

Research has demonstrated that OA can coexist with other inflammatory types of arthritis, which may sometimes remain undetected while a patient fails to achieve satisfactory treatment outcomes.^6^, ^7^, ^8^ The presence of calcium crystals is known to contribute to OA, and their inflammatory properties may mediate detrimental effects through innate immunity signaling pathways.^8^, ^9^ Septic arthritis can coexist with OA and crystal arthropathy (CPPD), which may present a diagnostic challenge.^10^, ^11^ Given the current limitations in effective therapies for nonsurgical treatment of OA, accurately distinguishing between primary OA and other arthritic conditions is crucial for effectively managing a patient's treatment plan. For instance, disease‐modifying antirheumatic drugs (DMARDs) have been shown to effectively treat inflammation‐mediated pain and slow disease progression in rheumatoid arthritis (RA) patients, but not in OA patients.^12^, ^13^, ^14^ Additionally, while arthroscopic irrigation has been shown to relieve pain in patients with crystalline arthritis, it is less effective for those with OA alone.^15^ There is a clear need for detection and differential diagnosis in the OA disease pathway to help avoid unnecessary treatments and enable more effective, tailored treatments for OA.

There are a variety of imaging techniques used in the diagnosis of OA such as radiographs, ultrasonographic imaging, and MRI, all with differing reliability and cost basis.^16^, ^17^, ^18^ The most widely used method for OA severity classification is Kellgren‐Lawrence score. However, it does not correlate well with the disease state until cartilage degeneration and joint morphology changes become visible on x‐ray, which is likely the beginning of end‐stage OA when treatment options are limited.^19^ Moreover, laboratory testing, especially blood testing, is not generally considered to be useful in the diagnosis of OA because inflammatory biomarkers are generally absent.^16^, ^20^, ^21^

Since OA is a joint disease, attention should be directed towards identifying joint‐specific biomarkers linked to its mechanical and biochemical pathogenesis. In the synovial fluid of the affected joint, Cartilage Oligomeric Matrix Protein (COMP) is known to be elevated when OA is present, and cartilage has degraded.^22^, ^23^ However, COMP alone is not an indicator of OA because other forms of arthritis also direct cartilage breakdown and release of COMP. By comparison, Interleukin‐8 (IL‐8) levels are low in OA and are elevated in cases of inflammatory arthritis, including rheumatoid arthritis (RA), crystal arthritis (CA), and native septic arthritis (NSA), or acute trauma.^24^, ^25^ Combining the results of these two biomarkers would enable diagnostic differentiation between primary OA and other inflammatory arthroses. Primary OA is unlikely when either COMP concentration or COMP/IL‐8 ratio in the synovial fluid is low since these conditions indicate either lack of cartilage degradation or presence of high inflammation. In contrast, a high COMP concentration result in combination with high COMP/IL‐8 ratio would be suggestive of low inflammation in the setting of cartilage deterioration, which is indicative of primary OA. Here, we report a two‐step test algorithm that combines the synovial fluid COMP and IL‐8 biomarker results to provide an accurate differential diagnosis of primary OA.

The purpose of this study was to address an unmet medical need to develop and validate a diagnostic test to provide physicians with an objective result from synovial fluid biomarkers indicative of primary OA. The diagnostic test can aid clinicians in clarifying the differential diagnosis of OA and ensure the evaluation of alternative or additional diagnoses, particularly in cases where the clinical presentation may not be straightforward.

## 2 MATERIALS AND METHODS

### 2.1 Specimen source and selection

This study was a retrospective cohort study (Level 3) using samples annotated with existing arthritis diagnoses. Patient knee synovial fluid samples were collected from an IRB‐approved clinical remnant sample source between 2016 and 2019 (OA samples – APSS‐44‐00, NCT02905240, CA/NSA samples – WIRB#20150222) or purchased (RA samples – BioIVT, Westbury, NY). Samples with insufficient volume or visually contaminated with blood were rejected, and a total of 171 specimens were used for the study. The study sample set consists of the following clinical arthritis diagnoses: 54 primary OA, 57 RA, 30 CA and 30 NSA. Primary OA diagnosis was defined as the presence of symptomatic early to moderate knee OA and an absence of severe OA, confirmed via radiograph as Kellgren‐Lawrence grades 2‐4, with exclusion of RA, gout, pseudogout, and other inflammatory arthritis or systemic inflammatory conditions as made by independent, expert clinicians and verified by independent, experienced clinical monitors. RA diagnosis was made by a licensed medical professional specializing in the care of patients with rheumatic conditions.^26^ CA diagnosis was assigned based on the presence/absence of MSU or CPPD crystals in the synovial fluid under polarized light microscopy (Table 1).^27^, ^28^ NSA diagnosis was assigned based on the results from the Synovasure® Alpha Defensin NSA Test, which includes Alpha Defensin and l‐lactate.^29^

**Table 1:** Characterization of synovial fluid sample crystal morphology in the determination of crystalline arthritis.

*Crystal Composition*	*Crystal Morphology*	*Light Polarization*		*Disorder*
		Direct	Compensated	
Monosodium Urate	Needle‐shaped	Highly birefringent	Parallel=yellow Perpendicular=blue	Gout
Calcium Pyrophosphate Dyhidrate	Rhombic/square	Weakly birefringent	Parallel=blue Perpendicular=yellow	CPPD* Disease

* Abbreviation: CPPD, Calcium pyrophosphate deposition (CPPD) disease has been formerly known as “pseudogout”.

**Figure 1:**
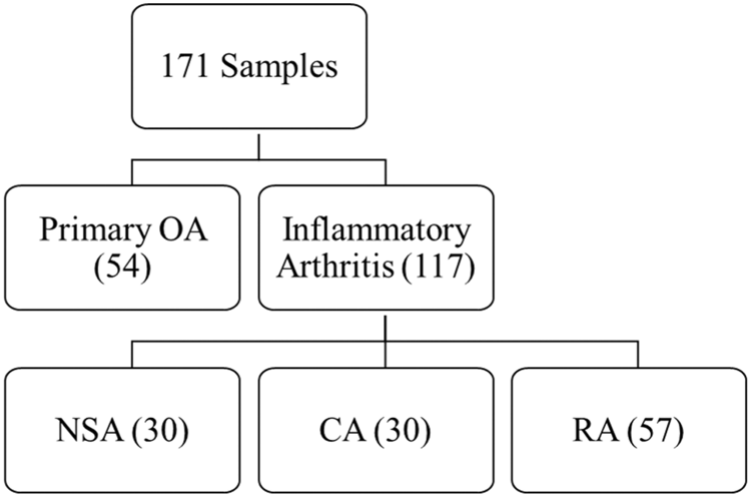
Sample cohort distribution used in the determination of the diagnostic accuracy of the OA dual‐biomarker algorithm. 171 samples used in the cohort consisted of 54 Primary OA and 117 Inflammatory Arthritis (NSA, CA, and RA). CA, Crystalline arthritis; NSA, Native Septic Arthritis; OA, Osteoarthritis; and RA, Rheumatoid arthritis.

All samples were randomized and blinded before testing. At the conclusion of testing, biomarker test results for each specimen were matched to the clinical diagnosis to determine the diagnostic performance. Figure 1 provides the summary of the specimen cohorts based on clinical diagnosis.

### 2.2 Specimen testing

All specimen testing was performed at CD Laboratories, Inc. (Zimmer Biomet, Towson, MD). Testing was conducted between the period of January 2020 to April 2020. Data analysis was performed at CD Diagnostics (Zimmer Biomet, Claymont, DE).

### 2.3 Specimen integrity

Specimen integrity testing was performed using internally established methods and reference intervals, based on spectrophotometric readings and cell count as previously described.^30^ Synovial fluid was diluted in buffer for spectrophotometer readings. This methodology was used to ascertain that samples are representative of synovial fluid that is not significantly diluted (saline) or contaminated (blood).

### 2.4 ELISA test for biomarkers (COMP and IL‐8)

A commercially available COMP ELISA test was used in the study (R&D Systems, Minneapolis, MN). The IL‐8 ELISA test was internally developed at CD Diagnostics (Zimmer Biomet, Claymont, DE). Both ELISA assays were separately validated for clinical synovial fluid testing at CD Laboratories. Samples were diluted to assay specifications with assay buffer before being used in the ELISA assays. No other sample manipulation was done. Test results were measured on the Biotek Epoch Microplate Spectrophotometer (Agilent Technologies) using Gen5 SW (v. 3.08.01).

### 2.5 Crystal evaluation

Crystal evaluation was performed by trained personnel following current standard microscopy techniques. The determination for monosodium urate (MSU) or calcium pyrophosphate dihydrate (CPPD) crystals are described in Table 1.

### 2.6 Data and statistical analysis

Biomarker concentration comparisons were done using a non‐parametric Mann‐Whitney test in Minitab 18. A 2 × 2 contingency table was used to evaluate the diagnostic accuracy of the primary OA algorithm. Sensitivity and specificity values were evaluated using MedCalc.^31^ Since equal weight was presumed for the sensitivity and specificity, a receiver operating characteristic (ROC) analysis was performed. Area under the curve (AUC) and Youden index (*J*) for the ROC curve analysis was generated. Subgroup comparisons were performed using Mood's Median Test. A one sample proportion with hypothesized proportion of 0.5 was used to evaluate gender proportions within cohorts.

## 3 RESULTS

### 3.1 Specimen integrity

All OA, CA, and NSA samples were within the internally established reference intervals. Three of 60 RA samples were outside of the reference intervals. They were therefore excluded from the study as diluted (*n* = 2) or contaminated (*n* = 1). The remaining 57 samples were included in the inflammatory arthritis cohort.

### 3.2 Baseline statistics

Mood's Median test assessing age within each cohort revealed that the population median from all arthritis cohorts were not equal (*p* = 0.002). In the OA cohort, the percentage of males and females were not significantly different (*p* = 0.892) at 52% and 48%, respectively. Similarly, the percentage of males and females in the RA and NSA cohorts were not significantly different. In the RA cohort, the males represented 37% and females represented 63% of the cohort (*p* = 0.063). In the NSA cohort, males represented 67%, and females represented 33% of the cohort (*p* = 0.099). However, in the CA cohort, males outnumbered the females significantly (87% vs 13%; *p* < 0.001) (Table 2).

**Table 2:** Demographics and COMP and IL‐8 concentrations and ratio among the different clinically diagnosed cohorts.

Clinical Cohort	*N*	Gender (%)		Median Age (IQR)	Samples with COMP > clinical decision limit N (%)	COMP concentration (ng/mL)	IL‐8 concentration (pg/mL)	COMP/IL‐8 Ratio	Samples Positive for OA Algorithm *N* (%)
OA	54	M	52	60 (55−65)	53 (98%)	3903.5 (2884.0−4610.0)	102.4 (102.4−459.0)	28.5	47 (87%)
		F	48						
CA	30	M	87	57 (49−72)	28 (93%)	3356 (2612.0−5211.0)	2187.8 (1320.0−5919.0)	1.6	7 (23.3%)
		F	13						
RA	57	M	37	69 (60−78)	24 (42%)	0.2 (0.2−2442.0)	175.3 (102.4−2768.0)	1.1	6 (10.5%)
		F	63						
NSA	30	M	67	67 (52−74)	3 (10%)	216 (79.0−859.0)	27598.8 (15,658.0−40,000.0)	0.1	0 (0%)
		F	33						

*Note*: IL‐8 concentration and COMP/IL‐8 ratio are reported for samples with COMP concentration above the clinical decision limit of 1500 ng/mL. Age and biomarker concentrations reported as median (IQR).

Abbreviations: CA, crystalline arthritis; COMP, cartilage oligomeric matrix protein; COMP, clinical decision limit is 1500 ng/mL; IL‐8, interleukin‐8; IQR, interquartile rang; NSA, native septic arthritis; OA, osteoarthritis; RA, rheumatoid arthritis.

### 3.3 Crystal examination

Samples with MSU crystals present were classified as “gout”. Samples with CPPD crystals present were classified as “CPPD.” Samples with no crystals present were classified as “no evidence of crystalline arthritis.” Crystalline bodies were found most often as singlet types (MSU, CPPD or cholesterol) and rarely in combination. Across the diagnostic cohorts, 2.3% of samples (4/171) were found to contain cholesterol crystals (all in the RA cohort). The total rate of MSU and CPPD crystal positivity was 40.9% (70 of 171), with MSU found in 24.0% (41/171), CPPD found in 16.4% (28/171), and both types of crystals found in <1% (1/171) (Supplementary Data, Table S2).

The CA cohort was composed of 30 specimens containing MSU or CPPD crystals and encompassed 30 of 70 (42.9%) samples identified with crystals (Table S2). The distribution of crystals for the remaining of the cohorts was as follows: 29 (41.4%) in RA, 11 (15.7%) in NSA, and no crystals (0%) in OA (Table S2). If OA and CA cohorts were omitted from the analysis due to pre‐selection for the absence or presence of crystals, respectively, the combined rate of crystal positivity in the NSA and RA cohorts was 50.6%.

For the purposes of clinical data analysis, samples were broadly classified as crystal arthritis (+) or (‐), regardless of which type of crystals were present in the specimen and location (extracellular or intracellular). The positive/negative classification did not take into account the Cholesterol crystals; however, presence of cholesterol crystals has been previously linked with rheumatoid arthritis.^32^

### 3.4 Osteoarthritis algorithm diagnostic accuracy

The OA algorithm (component of Synovasure® RISC™ Panel, CD Laboratories, Towson, MD) was designed to be indicative of primary OA when COMP concentration AND the COMP/IL‐8 ratio met the established decision thresholds (Supplementary Data, Figure S1). Samples with COMP concentration and COMP/IL‐8 ratio above the respective clinical decision limits were classified as primary OA positive. Samples with COMP concentration below the clinical decision limit were classified as primary OA negative, indicating an absence of measurable cartilage deterioration. Samples with COMP concentration above the clinical decision limit but COMP/IL‐8 ratio below the clinical decision limit were classified as primary OA negative, indicating potential secondary OA. The distribution of COMP concentration and ratios among the different clinically diagnosed cohorts are shown in Table 2.

Within the OA cohort, 47 out of the 54 specimens reported positive results on the OA algorithm (Table 3). There was only one specimen with COMP concentration below the clinical decision limit for which the ratio was not calculated. For the other 6 discordant samples, COMP met the threshold for cartilage damage with levels above the clinical decision limit. However, median IL‐8 concentration for these 7 specimens was significantly higher than the median concentrations reported in the other 47 OA specimens, resulting in a decreased COMP/IL‐8 ratio (Supplementary Data, Table S1). The median IL‐8 concentration for the primary OA‐positive specimens was at the lower limit of quantitation for the IL‐8 ELISA (102.4 pg/mL; Interquartile Range (IQR): 102.4−208.8), whereas the median IL‐8 concentration for the 7 negative samples was 1740.3 pg/mL (IQR: 1,378.0−2,959.2). A Mann‐Whitney test demonstrates a significant difference in IL‐8 concentration between the OA‐positive specimens and the OA‐negative specimens (*p* < 0.001). The median COMP concentration for the primary OA‐positive specimens was 3964.0 ng/mL (IQR: 3288.0−4686.0), whereas the median COMP concentration for the 7 negative samples was 2786.0 ng/mL (IQR: 1633.0−3920.0). A Mann‐Whitney test also demonstrates a significant difference between the OA‐positive and OA‐negative COMP values (*p* = 0.032). A detailed review of the seven subjects with low COMP/IL‐8 ratios in terms of demographic, age, or other observations showed that one patient presented with a Baker's cyst, which was drained in conjunction with the joint aspiration, likely contributing to the elevated IL‐8 (Supplementary Data, Table S1).

**Table 3:** 2 × 2 contingency table for assessment of the OA algorithm for differential diagnosis of primary OA.

	Condition Positive	Condition Negative
**Test Positive**	47	13
**Test Negative**	7	104

*Note*: Out of the *n* = 54 OA samples, 7 samples tested negative in the OA algorithm. For the *n* = 117 non‐OA samples, 13 samples tested positive for the OA algorithm.

Thirteen (13) samples from the non‐OA cohort tested positive on the OA algorithm and, of these, seven (7) were in the CA cohort and 6 were in the RA cohort. A 2 × 2 contingency table was constructed to evaluate diagnostic accuracy of the OA algorithm for differential diagnosis of primary OA versus inflammatory arthritis cohorts (Table 3). The values displayed in the contingency table were inputted into the diagnostic test calculator (MedCalc Version 20.109^31^) for analysis.

The primary OA diagnostic algorithm demonstrated clinical sensitivity of 87.0% (95% CI: 75.1% to 94.6%) and specificity of 88.9% (95% CI: 81.8% to 94.0%) for an overall accuracy of 88.3% (95% CI: 82.5% to 92.7%). The positive and negative predictive values (PPV and NPV) were 78.3% (95% CI: 68.2% to 85.9%) and 93.7% (95% CI: 88.1% to 96.8%), respectively. The positive likelihood ratio (LR+) was 7.8, and negative likelihood ratio (LR‐) was 0.15. Subgroup analysis was performed to evaluate the diagnostic sensitivity of the OA algorithm across KL‐grades, and consistent performance was observed (Table 4). Biomarker concentrations within each KL‐grade subgroup revealed that COMP concentration remains stable, whereas IL‐8 concentration is increased in the KL‐4 cohort, resulting in a significantly lower (*p* = 0.001) median COMP/IL‐8 ratio of 12.5, compared to 25.8 and 35.1 in the KL‐2 and KL‐3 subgroups, respectively **(**Supplementary Data, Table S3).

**Table 4:** OA Algorithm Diagnostic Sensitivity by KL Grade.

KL Grade	N	Sensitivity	95% CI
2	9	88.9%	51.7%–99.7%
3	25	88.0%	68.8%–97.5%
4	20	85.0%	62.1%–96.8%

Abbreviations: CI, confidence interval; KL, Kellgren‐Lawrence grade; OA, osteoarthritis.

**Figure 2:**
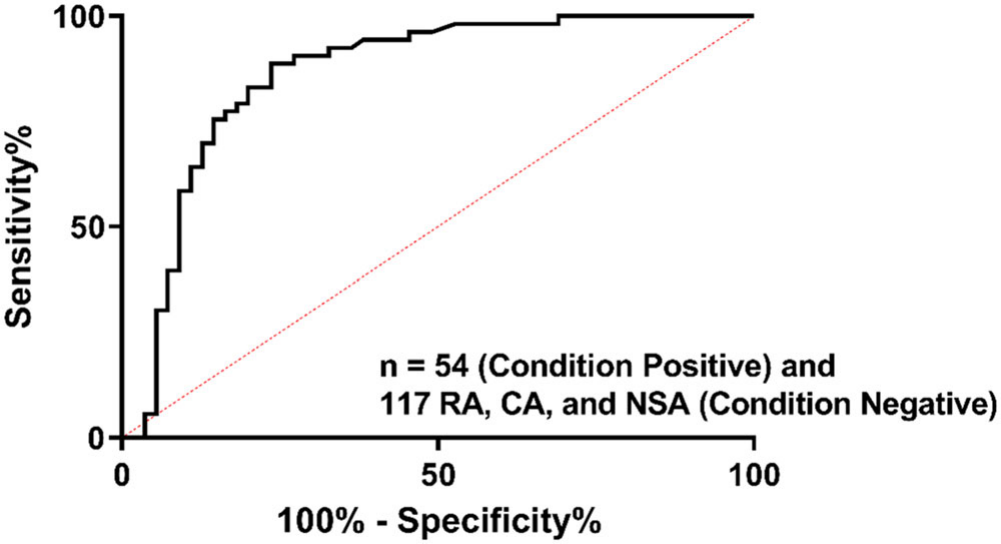
Receiver operating characteristic (ROC) Curve for the COMP/IL‐8 Dual‐Biomarker Algorithm for Osteoarthritis (OA algorithm). Condition positive samples are Osteoarthritis samples. Condition negative samples include RA, CA, and NSA samples. CA, Crystalline arthritis; NSA, Native septic arthritis; RA, Rheumatoid arthritis.

An analysis of the receiving operating characteristic (ROC) curve was derived for the OA algorithm ratios calculated on the primary OA cohort against the inflammatory arthritis cohort. (Figure 2). The results showed that the OA algorithm had an area under the curve (AUC) of 0.93 (95% CI: 0.89 to 0.97).

## 4 DISCUSSION

The baseline analysis of demographics between cohorts for age and within cohorts for gender indicated that, although there were significant differences observed, the cohorts are representative of the general population. For example, the older age of RA patients in this study are consistent with the recent literature reporting that the peak age at which RA presents has shifted from 50 to 59 to 60−69 in the past decade.^33^ OA, CA and NSA as cited in literature are widely reported in patients mostly over 50, which is also consistent with our study cohorts.^5^, ^34^, ^35^ Similarly, the higher percentage of males than females observed in the CA cohort is supported by the literature, as it is known that CA incidence is disproportionately more prevalent in men.^36^

Based on the characteristics of native knee samples received for clinical testing at CD Laboratories, the anticipated OA disease prevalence in the intended population was estimated to be 60%. When the data was adjusted for 60% disease prevalence, the accuracy of the OA algorithm was 87.8% (95% CI: 81.9% to 92.3%), with positive and negative predictive values of 92.2% (95% CI: 87.5% to 95.2%) and 82.1%, (95% CI: 69.6% to 90.2%), respectively.

As per guidelines to classify the accuracy of a diagnostic test, an AUC of 0.93 indicates that the OA algorithm demonstrates excellent ability to discriminate between primary OA and other forms of arthritis which may or may not be associated with secondary OA.^37^ Similarly, the algorithm's highly accurate diagnostic performance was exhibited by Youden's index (*J*) of 0.76. The test has a high LR+ of 7.8, suggesting an individual with osteoarthritis is 7.8 times more likely to render a positive result using this diagnostic algorithm than an individual with an inflammatory arthritis type (RA, CA, or NSA). Conversely, the LR‐ of 0.15 is very low, indicating an individual with inflammatory arthritis is about 7 times more likely to render a negative result using this diagnostic algorithm than an individual with primary OA.

In this study, a clear differentiation between primary OA and other comorbid arthrosis has been demonstrated. Previous studies have established that OA can coexist with other inflammatory arthritis types, which may sometimes remain undetected while a patient fails to achieve satisfactory treatment outcomes.^6^, ^7^, ^8^ The development of new treatment options and appropriate use of existing treatment options is dependent on an accurate and complete diagnosis of the disease. If presence of inflammatory arthritis remains undetected and untreated when the patient is advanced to surgical intervention, risk of postoperative complications, including periprosthetic joint infection (PJI), is increased.^38^, ^39^, ^40^ Additionally, delayed accurate diagnosis not only leads to delayed treatment, but also an increased economic cost not just to the patient, but also to the healthcare sytem.^41^ New tools such as delineating primary OA from other inflammatory joint conditions informs treatment decisions and enables optimization before surgical intervention to improve the prognosis.

When the OA algorithm is used as a diagnostic panel component in conjunction with other relevant synovial fluid biomarker test results, such as cell count with differential, crystal analysis, and tests for RA (rheumatoid factor (RF) and anti‐cyclic citrullinated peptide (anti‐CCP)), a much clearer picture of comorbidities, underlying inflammatory pathogenesis, or alternative diagnoses can emerge. When primary or secondary OA is indicated by the COMP/IL‐8 ratio, radiographs can be used to visualize the extent of structural deterioration in the joint, thereby further informing the most appropriate treatment decision. Incorporating the inflammatory status for each condition (from lowest to highest inflammation: OA < RA < active crystal disease < infection) facilitates risk stratification during disease triage or patient optimization before surgical intervention. Furthermore, being able to differentiate between the arthritic diseases would allow physicians to more confidently consider treatment options. For example, using disease modifying anti‐rheumatic drugs (DMARDs) for RA in the absence of biomarkers for other disease states; or, in the case of primary OA, nonsurgical intervention therapies such as orthobiologics, hyaluronic acid, and corticosteroids may gain stronger consideration when inflammatory disease‐related biomarkers remain undetected.

This study does have limitations, including source of samples resulting in potentially incomplete characterization. For example, RA samples were sourced from a biovendor and were not provided with any diagnosis‐confirming data, such as positive serum results for RF or anti‐CCP. Although a high prevalence of gout and CPPD crystals (50.9%) and evidence of native septic arthritis (21.1%) were observed in that cohort, the presence of these additional inflammatory comorbidities did not affect the study outcome. Given the observed comorbidities in the RA cohort, further evaluation to study the potential for misdiagnosis or underdiagnosed comorbidities may be warranted. Another limitation is that no consensus method for diagnosis of native septic arthritis exists, and so a previously validated laboratory‐developed algorithm was used for this purpose. These challenges are not unique to this study. Most deidentified remnant biological fluid samples obtained for research are not fully characterized. These samples may have various unknown comorbidities and even an altered biomarker profile because of uncontrolled events, such as timing of aspiration after medication use or exercise. Nonetheless, this represents a real‐world sample set and may even serve to further validate the robustness of the diagnostic performance achieved in this study. Although this study advances understanding of and offers a new tool for aiding the differential diagnosis of primary OA or revealing an underlying inflammatory arthropathy, it does not address the unmet need for accurate diagnosis of pre‐radiographic OA. Further research is needed to determine if the COMP/IL‐8 OA algorithm may prove to be useful in this setting.

Looking forward, differentiating primary OA from other inflammatory arthritis types contributes towards better diagnosis that enables accurate, and targeted treatment of primary OA. When coupled with other synovial fluid biomarker test results, this diagnostic algorithm also objectively reveals the relative inflammatory status of the joint based on the presence of inflammatory arthroses.

## AUTHOR CONTRIBUTIONS

Daniel Keter, Van Thai‐Paquette, John Miamidian, and Krista Toler contributed to the design, analysis, and interpretation of the data and to the writing and revision of the manuscript. Simmi Gulati contributed to the acquisition of data. All authors have read and approved the final submitted manuscript.

## Supporting information

Supporting information.

Supporting information.

Supporting information.

Supporting information.

Supporting information.

Supporting information.

Supporting information.

Supporting information.

Supporting information.

Supporting information.

Supporting information.

Supporting information.

Supporting information.

Supporting information.

Supporting information.

Supporting information.

Supporting information.

Supporting information.

Supporting information.

Supporting information.

Supporting information.

Supporting information.

Supporting information.
