# Supplementary material for: Synovial fluid dual‐biomarker algorithm accurately differentiates osteoarthritis from inflammatory arthritis
Source: J Orthop Res. 2024 Dec 18;43(2):304–10. doi: 10.1002/jor.26005 (PMC11701394; doi:10.1002/jor.26005)
Supplement: Supplementary file 1 — Supporting information. [file JOR-43-304-s023.docx]

**Supplementary Data**

An algorithm was developed (**Figure S1**) using COMP concentration and dual biomarker algorithm values to accurately differentiate primary OA from other inflammatory arthritis types.

**A B**


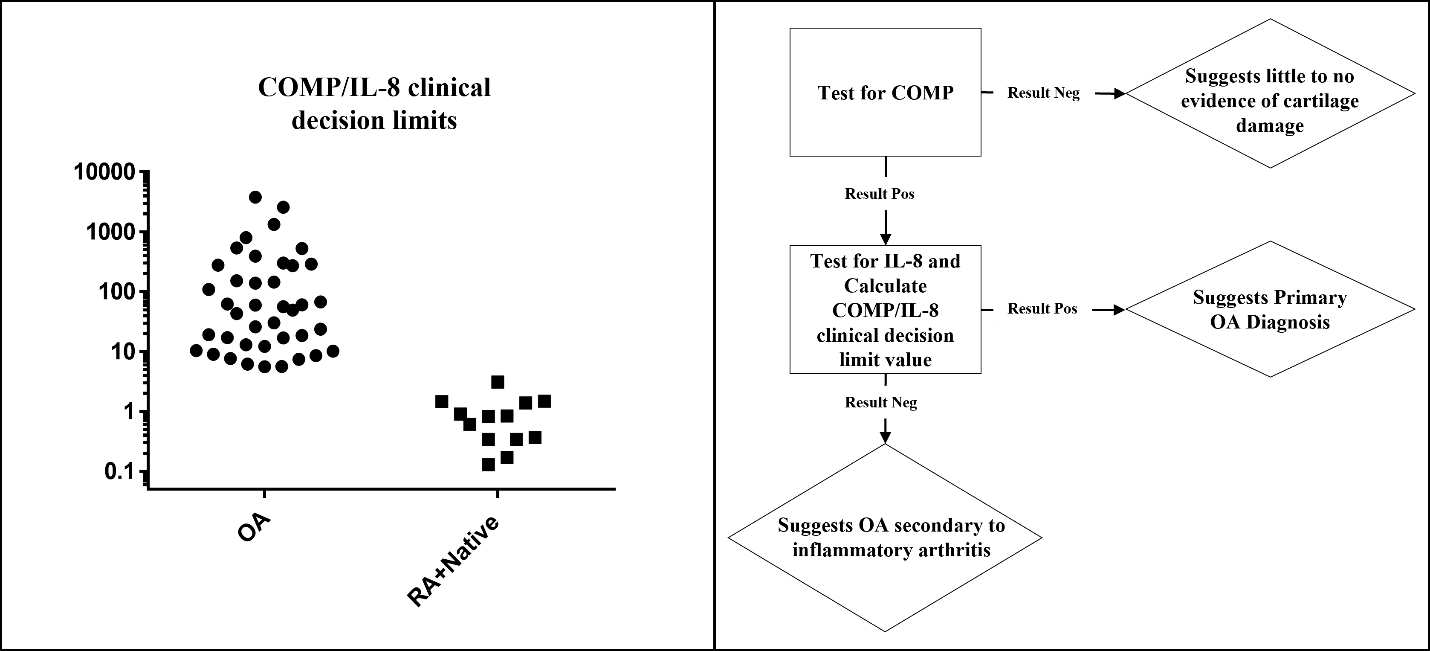


**FIGURE S1.** (A) Discovery data demonstrating the utility of COMP and IL-8 as a dual biomarker algorithm in differentiating osteoarthritis from other arthropathies. (B) Algorithm decision outline to determine whether samples were positive for primary OA.

**TABLE S1.** Discordant COMP/IL-8 found in 7 samples of OA cohort.

| Sample | COMP (ng/mL) | IL-8 (pg/mL) | COMP/IL-8 (if COMP > clinical decision limit values) | Demographics and OA Stage | Other Comments/ NTFs Regarding Eligibility and Procedure |
| --- | --- | --- | --- | --- | --- |
|  |  |  |  |  |  |
| 1 | 1,675 | 2,089 | 0.8 | Female | This was a Baker’s Cyst subject in which we allowed for the cyst drainage is conjunction with the joint aspiration/ injection. |
|  |  |  |  | African American |  |
|  |  |  |  | Age 59 |  |
|  |  |  |  | BMI 32, KL 2 |  |
| 2 | 3,920 | 1,531 | 2.6 | Male | No noted issues with aspiration of synovial fluid or injection. |
|  |  |  |  | White (non-Hispanic) |  |
|  |  |  |  | Age 63 |  |
|  |  |  |  | BMI 25, KL 4 |  |
| 3 | 3,890 | 2,959 | 1.3 | Male | No noted issues with aspiration of synovial fluid or injection. |
|  |  |  |  | White (non-Hispanic) |  |
|  |  |  |  | Age 56 |  |
|  |  |  |  | BMI 37, KL 4 |  |
| 4 | 4,101 | 1,378 | 3.0 | Female | No noted issues with aspiration of synovial fluid or injection. |
|  |  |  |  | White (non-Hispanic) |  |
|  |  |  |  | Age 57 |  |
|  |  |  |  | BMI 31, KL 3 |  |
| 5 | 1,633 | 472 | 3.5 | Female | No noted issues with aspiration of synovial fluid or injection. |
|  |  |  |  | White (non-Hispanic) |  |
|  |  |  |  | Age 52 |  |
|  |  |  |  | BMI 40, KL 3 |  |
| 6 | 2,786 | 1,740 | 1.6 | Male | No noted issues with aspiration of synovial fluid or injection. |
|  |  |  |  | White (non-Hispanic) |  |
|  |  |  |  | Age 65 |  |
|  |  |  |  | BMI 27, KL 4 |  |
| 7 | 1,420 | 3,954 | NIL | Male | No noted issues with aspiration of synovial fluid or injection. |
|  |  |  |  | White (non-Hispanic) |  |
|  |  |  |  | Age 77, KL 3 |  |

COMP – Cartilage Oligomeric Matrix Protein; IL-8 – Interleukin-8; KL – Kellgren-Lawrence; BMI – Body Mass Index; NTF – Note To File

**TABLE S2.** Distribution of crystal arthritis in study cohorts. Percentages reflect the proportion of samples with the crystal type to the total number of samples (%) in the clinical cohort (NSA = 30, CA = 30, RA = 57, OA = 54).

|  | **Crystal Type** | | | |
| --- | --- | --- | --- | --- |
| Clinical  Cohort | Monosodium urate (MSU) | Calcium pyrophosphate dihydrate (CPPD) | MSU AND CPPD | Cholesterol |
| NSA | 2(6.7%) | 9(30%) | 0(0%) | 0(0%) |
| RA | 25(43.9%) | 4(7%) | 0(0%) | 4(7%) |
| OA | 0(0%) | 0(0%) | 0(0%) | 0(0%) |
| CA | 14(46.7%) | 15(50%) | 1(3.3%) | 0(0%) |
| Total | 41 | 28 | 1 | 4 |

NSA = native septic arthritis, RA = rheumatoid arthritis, OA = osteoarthritis, CA = crystalline arthritis, MSU = monosodium urate, CPPD = calcium pyrophosphate deposition.

**TABLE S3.** Subgroup Comparison of COMP and IL-8 concentrations and COMP/IL-8 ratio between KL Grades 2-4.

| Variable | KL  Grade | N | Median | IQR |
| --- | --- | --- | --- | --- |
| COMP (ng/mL) | 2 | 9 | 3,321 | 2,160-4,103 |
|  | 3 | 25 | 3995 | 3,346-4,772 |
|  | 4 | 20 | 3905 | 2,732-4,361 |
| IL-8 (pg/mL) | 2 | 9 | 102.4 | 102.4-331.9 |
|  | 3 | 25 | 102.4 | 102.4-280.0 |
|  | 4 | 20 | 236.7 | 102.4-696.2 |
| COMP/IL-8 ratio  (if COMP>1500ng/mL) | 2 | 9 | 25.8 | 9.7-37.2 |
|  | 3 | 24 | 35.1 | 26.0-44.1 |
|  | 4 | 20 | 12.5 | 6.3-27.7 |

COMP – Cartilage Oligomeric Matrix Protein; IL-8 – Interleukin-8; KL – Kellgren-Lawrence; IQR – Interquartile Range
