# Supplementary material for: Synovial fluid dual‐biomarker algorithm accurately differentiates osteoarthritis from inflammatory arthritis
Source: J Orthop Res. 2024 Dec 18;43(2):304–10. doi: 10.1002/jor.26005 (PMC11701394; doi:10.1002/jor.26005)
Supplement: Supplementary file 7 — Supporting information. [file JOR-43-304-s002.pdf]

Western Institutional Review Board®
1019 39th Avenue SE Suite 120 | Puyallup, WA 98374-2115
Office: (360) 252-2500 | **Toll Free**: (800) 562-4789
www.wirb.com

Certificate of Approval

THE FOLLOWING WERE APPROVED

INVESTIGATOR: Robert Magnussen MD, MPH
2050 Kenny Road
Pavilion Suite 3100
Columbus, Ohio 43221

BOARD ACTION DATE: 10/28/2016
PANEL: 2
STUDY APPROVAL EXPIRES: 09/30/2017
STUDY NUM: 1169035
WIRB PRO NUM: 20161893
ONLINE TRACKING: 11-1695725
INVEST NUM: 215539
WO NUM: 1-966466-1
CONTINUING REVIEW: Annually
SITE STATUS REPORTING: Annually
INST. NUM: 2016W0165

SPONSOR: Biomet Biologics, LLC
PROTOCOL NUM: APSS-44-00
AMD. PRO. NUM:
TITLE:
A Multicenter, Double-Blind, Randomized, Saline-Controlled Study of a Single, Intra-Articular Injection of Autologous Protein Solution in Patients with Knee Osteoarthritis

APPROVAL INCLUDES:

IF YOU HAVE ANY QUESTIONS, CONTACT WIRB AT 1-800-562-4789
This is to certify that the information contained herein is true and correct as reflected in the records of the Western Institutional Review Board (WIRB), OHRP/FDA parent organization number IORG 0000432, IRB registration number IRB00000533. WE CERTIFY THAT WIRB IS IN FULL COMPLIANCE WITH GOOD CLINICAL PRACTICES AS DEFINED UNDER THE U.S. FOOD AND DRUG ADMINISTRATION (FDA) REGULATIONS, U.S. DEPARTMENT OF HEALTH AND HUMAN SERVICES (HHS) REGULATIONS, AND THE INTERNATIONAL CONFERENCE ON HARMONISATION (ICH) GUIDELINES.

Copyright © 2013 Western Institutional Review Board, Inc. All rights reserved.

Investigator
Administrative Letter (09-21-2016) Pregnancy Test Requirement
Adverse Event Form #14999517.0 - As Submitted
Advertisement - Answering Message Script - Thank you for your interest #15143272.0 - As Submitted
Advertisement - Clinical Trials Fact Sheet - What Is A Clinical Trial #14858799.0 - As Submitted
Advertisement - Patient Education Brochure - Progress IV Clinical Trial Patient #14858797.1 - As Submitted
Advertisement - Patient FAQ - Progress IV Clinical Trial FAQ #14858803.1 - As Submitted
Advertisement - Pre-Screening Message Content - Thanks for your interest in #15143274.0 - As Submitted
Advertisement - Social Media Posts - Working in the yard a #14858795.1 - As Submitted
Advertisement - Table Topper - If You Have Knee OA #14858805.0 - As Submitted
Advertisement - Trial Site Video Recording - Osteoarthritis (OA) the most common type #14858793.1 - As Submitted
Advertisement - Website Copy (Patients) - OA of the knee is #14858794.1 - As Submitted
Clinical Trial Fact Sheet #14858804.0 - As Submitted
Clinical Trial FAQ for Health Care Providers #14858800.0 - As Modified
Demographics and Medication Use - Screening Visit #14999525.0 - As Submitted
Eligibility Criteria - Screening Visit #14999521.0 - As Submitted
EQ-5D Health Questionnaire - 2nd Injection Follow-Up Visit #14999572.0 - As Submitted
EQ-5D Health Questionnaire - Injection Visit #14999529.0 - As Submitted
EQ-5D Health Questionnaire - Month 1 Visit #14999535.0 - As Submitted
EQ-5D Health Questionnaire - Month 12 Visit #14999555.0 - As Submitted
EQ-5D Health Questionnaire - Month 3 Visit #14999540.0 - As Submitted
EQ-5D Health Questionnaire - Month 6 Visit #14999550.0 - As Submitted
Follow-Up Visit - 2nd Injection Follow-Up Visit #14999570.0 - As Submitted
Follow-Up Visit - Month 1 Visit #14999533.0 - As submitted
Follow-Up Visit - Month 12 Visit #14999552.0 - As Submitted
Follow-Up Visit - Month 3 Visit #14999537.0 - As Submitted
Follow-Up Visit - Month 6 Visit #14999543.0 - As Submitted
Injection Visit #14999528.0 - As Submitted
Injection Visit 2 - Open-Label 2nd Injection Visit #14999557.0 - As Submitted
Monitoring Plan #14999499.0 - As Submitted
Osteoarthritis Fact Sheet #14858802.0 - As Submitted
Pocket Brochure - Schedule of Visits and Inclusion-Exclusion Criteria #14858796.0 - As Submitted
Protocol (06-28-2016) Version 1.0
Protocol Deviation Form #14999518.0 - As Submitted
Sample Processing - Injection Visit #14999532.0 - As Submitted
Statistical Analysis Plan #14999497.0 - As Submitted
Study Exit Form #14999520.0 - As Submitted
VAS Pain Assessment - 2nd Injection Follow-Up Visit #14999576.0 - As Submitted
VAS Pain Assessment - Injection Visit #14999531.0 - As Submitted
VAS Pain Assessment - Month 1 Visit #14999536.0 - As Submitted
VAS Pain Assessment - Month 12 Visit #14999556.0 - As Submitted
VAS Pain Assessment - Month 3 Visit #14999541.0 - As Submitted
VAS Pain Assessment - Month 6 Visit #14999551.0 - As Submitted
Website Copy - Health Care Providers #14858801.1 - As Submitted
WOMAC Osteoarthritis Index LK 3.1 - 2nd Injection Follow-Up Visit #14999571.0 - As Submitted
WOMAC Osteoarthritis Index LK 3.1 - Month 1 Visit #14999534.0 - As Submitted
WOMAC Osteoarthritis Index LK 3.1 - Month 12 Visit #14999554.0 - As Submitted
WOMAC Osteoarthritis Index LK 3.1 - Month 3 Visit #14999539.0 - As Submitted
WOMAC Osteoarthritis Index LK 3.1 - Month 6 Visit #14999548.0 - As Submitted
WOMAC Osteoarthritis Index LK 3.1 - Screening Visit #14999524.0 - As Submitted
Consent Form [IN0]

WIRB APPROVAL IS GRANTED SUBJECT TO:
The Board requires that all subjects must be able to consent for themselves to be enrolled in this study. This means that you cannot enroll incapable subjects who require enrollment by consent of a legally authorized representative.

WIRB HAS APPROVED THE FOLLOWING LOCATIONS TO BE USED IN THE RESEARCH:

The Ohio State University, 2050 Kenny Road, Pavilion Suite 3100, Columbus, Ohio 43221
OSU Hospital East, 1492 E. Broad Street, Columbus, Ohio 43205

If the PI has an obligation to use another IRB for any site listed above and has not submitted a written statement from the other IRB acknowledging WIRB's review of this research, please contact WIRB's Client Services department.

Copyright © 2011 Western Institutional Review Board, Inc. All rights reserved.

ALL WIRB APPROVED INVESTIGATORS MUST COMPLY WITH THE FOLLOWING:

1. Conduct the research in accordance with the protocol, applicable laws and regulations, and the principles of research ethics as set forth in the Belmont Report.

2. Although a participant is not obliged to give his or her reasons for withdrawing prematurely from the clinical trial, the investigator should make a reasonable effort to ascertain the reason, while fully respecting the participant's rights.

3. Unless consent has been waived, conduct the informed consent process without coercion or undue influence, and provide the potential subject sufficient opportunity to consider whether or not to participate. (Due to the unique circumstances of research conducted at international sites outside the United States and Canada, when there is a local IRB and WIRB approved materials are reviewed by the local IRB and translated into the local language, the following requirements regarding consent forms bearing the WIRB approval stamp and regarding certification of translations are not applicable.)
    a. Use only the most current consent form bearing the WIRB "APPROVED" stamp.
    b. Provide non-English speaking subjects with a certified translation of the approved consent form in the subject's first language. The translation must be approved by WIRB unless other arrangements have been made and approved by WIRB.
    c. Obtain pre-approval from WIRB for use of recruitment materials and other materials provided to subjects.

4. Enrollment of limited readers and non-readers: unless consent has been waived or the protocol excludes enrollment of limited readers or non-readers, involve an impartial witness in the consent process when enrolling limited or non-readers and document the participation of the impartial witness using the designated signature lines on the WIRB-approved consent form. In the absence of designated signature lines, download the WIRB standard impartial witness form from www.wirb.com.

5. Obtain pre-approval from WIRB for changes in research.

6. Obtain pre-approval from WIRB for planned deviations and changes in research activity as follows:
    - If the research is federally funded, conducted under an FWA, or is a clinical investigation of a drug or biologic, then all planned protocol deviations must be submitted to WIRB for review and approval prior to implementation except where necessary to eliminate apparent immediate hazards to the human subjects [(DHHS 45 CFR § 46.103(b)(4); (FDA 21 CFR § 56.108(a)(4); ICH 3.3.7].
    - However, if the research is a clinical investigation of a device and the research is not federally funded and not conducted under an FWA, then only planned protocol deviations that may adversely affect the rights, safety or welfare of subjects or the integrity of the research data should be submitted to WIRB for review and approval prior to implementation except where necessary to eliminate apparent immediate hazards to the human subjects [(DHHS 45 CFR § 46.103(b)(4); (FDA 21 CFR § 56.108(a)(4); ICH 3.3.7].

    The reason for these different requirements regarding planned protocol deviations is that the Office for Human Research Protections (OHRP) and the Food and Drug Administration (FDA) drug and biologic divisions have adopted the regulatory interpretation that every planned protocol deviation is a change in research that needs prior IRB review and approval before implementation; however, the FDA device division operates under a distinct regulation (See 21 CFR 812.150(a)(4).

    Deviations necessary to eliminate apparent immediate hazards to the human subjects should be reported within 10 days.

7. Report the following information items to the IRB within 5 days:
    a. New or increased risk
    b. Protocol deviation that harmed a subject or placed subject at risk of harm
    c. Protocol deviation made without prior IRB approval to eliminate an immediate hazard to a subject
    d. Audit, inspection, or inquiry by a federal agency
    e. Written reports of federal agencies (e.g., FDA Form 483)
    f. Allegation of Noncompliance or Finding of Noncompliance
    g. Breach of confidentiality
    h. Unresolved subject complaint
    i. Suspension or premature termination by the sponsor, investigator, or institution
    j. Incarceration of a subject in a research study not approved to involve prisoners
    k. Adverse events or IND safety reports that require a change to the protocol or consent
    l. State medical board actions
    m. Unanticipated adverse device effect
    n. Information where the sponsor requires prompt reporting to the IRB

    Information not listed above does not require prompt reporting to WIRB.

Copyright © 2011 Western Institutional Review Board, Inc. All rights reserved.

Please go to www.wirb.com for complete definitions and forms for reporting.

8. Provide reports to WIRB concerning the progress of the research, when requested.

9. Ensure that prior to performing study-related duties, each member of the research study team has had training in the protection of human subjects appropriate to the processes required in the approved protocol.

Federal regulations require that WIRB conduct continuing review of approved research. You will receive Continuing Review Report forms from WIRB. These reports must be returned even though your study may not have started.

DISTRIBUTION OF COPIES:
Contact, Company
Angela Pedroza, The Ohio State University
Main Contact OSURF, The Ohio State University Research Foundation
Stephanie Hulec, IMARC Research Inc
Carleton Southworth, Biomet Biologics LLC
Robert Magnussen MD, MPH, The Ohio State University

 Copyright © 2011 Western Institutional Review Board, Inc. All rights reserved.
