# Supplementary material for: Synovial fluid dual‐biomarker algorithm accurately differentiates osteoarthritis from inflammatory arthritis
Source: J Orthop Res. 2024 Dec 18;43(2):304–10. doi: 10.1002/jor.26005 (PMC11701394; doi:10.1002/jor.26005)
Supplement: Supplementary file 9 — Supporting information. [file JOR-43-304-s010.pdf]

PARTNERS HEALTHCARE
FOUNDED BY BRIGHAM AND WOMEN'S HOSPITAL AND MASSACHUSETTS GENERAL HOSPITAL

Partners Human Research Committee
399 Revolution Drive, Suite 710
Somerville, MA 02145
Tel: (857) 282-1900
Fax: (857) 282-5693

Initial Review: Notification of IRB Approval/Activation
Protocol #: 2016P001958/BWH

Date: November 2, 2016

To: Andreas H Gomoll, MD
BWH
Orthopedics

From: Partners Human Research Committee
399 Revolution Drive, Suite 710
Somerville, MA 02145

Title of Protocol:	A Multicenter, Double-Blind, Randomized,Saline-Controlled Study of a Single, Intra-Articular Injection of Autologous Protein Solution in Patients with Knee Osteoarthritis
Version Date:	6/28/2016
Sponsor/Funding Support:	
	Proposal Title: CTA: APSS-44-00<br>Name: Biomet Biologics LLC<br>Sponsor Number: APSS-44-00
	Name: Biomet Biologics, LLC

Study Population:	Adults
Consent/Authorization:	Required
Documentation of Consent:	Written
Informed Consent From:	Adult Subject
Informed Consent By:	Licensed Physician Investigator
IRB Review Type:	Full
IRB Approval Date:	10/28/2016
Approval Activation Date:	11/2/2016
IRB Expiration Date:	10/28/2017

This project has been reviewed by BWH IRB . During the review of this project, the IRB specifically considered (i) the risks and anticipated benefits, if any, to subjects; (ii) the selection of subjects; (iii) the procedures for obtaining and documenting informed consent; (iv) the safety of subjects; and (v) the privacy of subjects and confidentiality of the data.

Please note that if an IRB member had a conflict of interest with regard to the review of this project, consistent with IRB policies and procedures, the member was required to leave the room during the discussion and vote on

Partners Human Research Committee
399 Revolution Drive, Suite 710
Somerville, MA 02145
Tel: (857) 282-1900
Fax: (857) 282-5693

this project except to provide information requested by the IRB.

The following protocol documents have been approved and supporting documents noted by the IRB:

- ***Protocol Summary, Version Date: June 28, 2016***
- ***Schema, Version Date: June 28, 2016***
- ***Detailed Protocol, Version Date: June 28, 2016***
- ***Consent Form (1)***
- ***Advertisements (11)***
- ***Flyer (1)***
- ***Recruitment Letter (1)***
- ***WOMAC LK 3.1 Questionnaire (1)***
- ***EQ-5D (1)***
- ***VAS Pain Scale (1)***
- ***IDE Approval Letter dated July 29, 2016***
- ***Investigator's Brochure, Sponsor Protocol Number: APSS-44-00, Version 1, Version Date: 28 June 2016***
- ***Protocol Supplement 1 – Package Insert – eSTRIDE® APS Kit***
- ***Protocol Supplement 2 - Sample Case Report Forms***
- ***Protocol Supplement 3 - Statistical Analysis Plan***
- ***Protocol Supplement 4 – Monitoring Plan for Protocol Number: APSS-44-00***

As Principal Investigator, you are responsible for ensuring that this project is conducted in compliance with all applicable federal, state and local laws and regulations, institutional policies, and requirements of the IRB, which include, but are not limited to, the following:

1. Submission of any and all proposed changes to this project (e.g., protocol, recruitment materials, consent form, status of the study, etc.) to the IRB for review and approval prior to initiation of the change(s), except where necessary to eliminate apparent immediate hazards to the subject(s). Changes made to eliminate apparent immediate hazards to subjects must be reported to the IRB as an unanticipated problem.
2. Submission of continuing review submissions for re-approval of the project prior to expiration of IRB approval and a final continuing review submission when the project has been completed.
3. Submission of any and all unanticipated problems, including adverse event(s) in accordance with the IRB's policy on reporting unanticipated problems including adverse events.
4. Obtaining informed consent from subjects or their legally authorized representative prior to initiation of research procedures when and as required by the IRB and, when applicable, documenting informed consent using the current IRB approved consent form(s)with the IRB-approval stamp in the document footer.
5. Informing all investigators and study staff listed on the project of changes and unanticipated problems, including adverse events, involving risks to subjects or others.
6. When investigator financial disclosure forms are required, updating your financial interests in Insight and for informing all site responsible investigators, co-investigators and any other members of the study staff identified by you as being responsible for the design, conduct, or reporting of this research study of their obligation to update their financial interest disclosures in Insight if (a) they have acquired new financial interests related to the study and/or (b) any of their previously reported financial interests related to the study have changed.

The IRB has the authority to terminate projects that are not in compliance with these requirements.
Questions related to this project may be directed to Deena G Segal, DSEGAL@PARTNERS.ORG, 857-282-1910.

CC: Amy Phan, BWH, Research Assistant
