# Supplementary material for: Synovial fluid dual‐biomarker algorithm accurately differentiates osteoarthritis from inflammatory arthritis
Source: J Orthop Res. 2024 Dec 18;43(2):304–10. doi: 10.1002/jor.26005 (PMC11701394; doi:10.1002/jor.26005)
Supplement: Supplementary file 10 — Supporting information. [file JOR-43-304-s001.pdf]

Florida Hospital
Institutional Review Board
901 N. Lake Destiny Road
Suite 400
Maitland, FL 32751
Telephone: (407) 303-5581
Fax: (407) 303-2567
FWA: 00002060

November 17, 2016

To: J. Dean Cole, MD

On November 17, 2016 the IRB approved the following through November 7, 2017 inclusive.

Review Type:	Expedited Review
Title:	A multicenter, double-blind, randomized, saline-controlled study of a single, intra-articular injection of autologous protein solution in patients with knee osteoarthritis - nSTRIDE APS
Principal Investigator:	J. Dean Cole, MD
IRB number:	870850-4
IND or IDE number, if any:	*17069*
Expedited Category:	MM1
Documents reviewed:	• Consent Form - 870850-APSS-44-00 ICF-FH Rev16Nov2016.docx (UPDATED: 11/17/2016)<br>• Cover Sheet - Response to Conditional Approval Letter.docx (UPDATED: 11/17/2016)

Before November 7, 2017, you are to submit a continuing review to request continuing approval or closure. If the IRB does not grant continuing review, approval of this protocol ends after November 7, 2017.

Copies of any approved consent documents, consent scripts, or assent documents are published.

In conducting this study, you are required to follow the requirements in "INVESTIGATOR GUIDANCE: Investigator Obligations (HRP-800)."

If you have any questions, please contact the Florida Hospital IRB at 407-303-5581 or FH.IRB.general@flhosp.org. Please include your project title and IRBNet ID number in all correspondence with this office.

Sincerely,

IRB Office

Generated on IRBNet
