# Supplementary material for: Synovial fluid dual‐biomarker algorithm accurately differentiates osteoarthritis from inflammatory arthritis
Source: J Orthop Res. 2024 Dec 18;43(2):304–10. doi: 10.1002/jor.26005 (PMC11701394; doi:10.1002/jor.26005)
Supplement: Supplementary file 11 — Supporting information. [file JOR-43-304-s006.pdf]

The University of Kansas Medical Center

Human Research Protection Program

APPROVAL OF SUBMISSION

November 17, 2016

Neil Segal
nsegal@kumc.edu

Dear Neil Segal:

On 11/1/2016, the IRB reviewed the following submission:

Type of Review:	Initial Study
Reviewing IRB:	IRB00006196
FWA#:	00003411
IRB#:	STUDY00140155
Title:	A Multicenter, Double-Blind, Randomized, Saline-Controlled Study of a Single, Intra-Articular Injection of Autologous Protein Solution in Patients with Knee Osteoarthritis
Investigator:	Neil Segal
Funding:	Name: Biomet Biologics, LLC
IND, IDE or HDE:	IDE or HDE #17069
Documents submitted for the above review:	• HSC Supplemental Application<br>• Scientific Merit Approval<br>• Radiation Safety Application<br>• Response to Modifications<br>• PROGRESS IV IDE Protocol APS-44-00 Version 1 dated 28JUN2016<br>• PROGRESS IV Investigator's Brochure dated 28JUN2016<br>• IDE 17069 Approval Letter 2016-07-29 For Distribution<br>• PROGRESS IV IDE Protocol Supplement 1 Package Insert dated 28JUN2016<br>• Sponsor's PROGRESS IV Informed Consent Template dated 28JUN2016<br>• KUMC Main CF MODS tracked<br>• KUMC Main CF MODS clean<br>• PROGRESS IV - HCP FAQ - 8.8.16<br>• PROGRESS IV - FAQ - Patient FAQ - 9.12.16<br>• PROGRESS IV - Clinical Trials Fact Sheet - 8.8.16<br>• PROGRESS IV - Trial Site Video Script - 8.8.16<br>• PROGRESS IV - Website - Patient Website Copy - 9.12.16<br>• PROGRESS IV - HCP Website Copy - 8.8.16<br>• PROGRESS IV - PROGRESS IV Clinical Trial Fact Sheet - 8.8.16<br>• PROGRESS IV - Osteoarthritis Fact Sheet - 8.8.16<br>• PROGRESS IV - Social Media - Social Media Posts - 9.14.16<br>• PROGRESS IV - Table Topper - 8.10.16<br>• PROGRESS IV - IE Pocket Brochure - 8.8.16<br>• PROGRESS IV - Patient Education Brochure - 8.8.16

The IRB approved this submission from 11/17/2016 to 11/16/2017 inclusive.

Your approved documents are stored in the "Documents" tab for this study in the eCompliance system. The IRB stamped consent form(s) can be found under the "Final" column on the right side of the screen. These are the **only** valid versions for documenting informed consent.

If continuing review approval is not granted on or before 11/16/2017, approval of this study expires after that date.

Approval of this research is contingent upon your agreement to:

(1) Adhere to all KUMC Policies and Procedures Relating to Human Subjects, as written in accordance with the Code of Federal Regulations (45 CFR 46).

(2) Ensure that all study personnel are adequately trained for their role on the study.

(3) Maintain current training in human subjects protection and current disclosure of conflicts of interest as required by KUMC policy.

(4) Except where informed consent and HIPAA authorization have been formally waived by the IRB, seek, document and maintain records of informed consent and HIPAA authorization from each prospective subject or his/her legally authorized representative.

(5) Maintain copies of all pertinent information related to the research study including, but not limited to, video and audio tapes, instruments, copies of written informed consent agreements, and any other supportive documents in accordance with the KUMC Research Records Retention Policy.

(6) Report adverse events, non-compliance and other problems to the IRB by submitting a Report of New Information.

(7) Follow the IRB-approved protocol. Submit Modifications to the IRB for any proposed changes from the previously approved project. Changes may not be initiated without prior IRB review and approval, unless a delay in implementation would place subjects at risk.

(8) Submit a Continuing Review to the KUMC IRB before the expiration date. Federal regulations and IRB policies require continuing review of research at intervals appropriate to the degree of risk, but not less than once per year.

For more information on Human Subjects Research Policies or using the eCompliance system, please see our website at: http://www.kumc.edu/compliance/human-research-protection-program/institutional-review-board.html

If you have any questions regarding the human subject protection process, please do not hesitate to contact our office at 913-588-1240 or humansubjects@kumc.edu.

Sincerely,
Karen Blackwell
