# Supplementary material for: Synovial fluid dual‐biomarker algorithm accurately differentiates osteoarthritis from inflammatory arthritis
Source: J Orthop Res. 2024 Dec 18;43(2):304–10. doi: 10.1002/jor.26005 (PMC11701394; doi:10.1002/jor.26005)
Supplement: Supplementary file 16 — Supporting information. [file JOR-43-304-s005.pdf]

UCLA OHRPP Office of the Human Research Protection Program

University of California Los Angeles
10889 Wilshire Blvd, Suite 830
Los Angeles, CA 90095-1406

http://ora.research.ucla.edu/ohrpp
General Campus IRB: (310) 825-7122
Medical IRB: (310) 825-5344

APPROVAL NOTICE
New Study

**DATE:**	6/21/2017
**TO:**	KRISTOFER JONES, MD<br>ORTHOPEDIC SURGERY
**FROM:**	DANIEL CLEMENS, MD, PhD<br><br>Chair, MIRB1
**RE:**	IRB#16-001442<br>A Multicenter, Double-Blind, Randomized, Saline-Controlled Study of a Single, Intra-Articular Injection of Autologous Protein Solution in Patients with Knee Osteoarthritis<br>Version: Original 06-28-16

The UCLA Institutional Review Board (UCLA IRB) has approved the above-referenced study. UCLA's Federalwide Assurance (FWA) with Department of Health and Human Services is FWA00004642.

Submission and Review Information

Type of Review	Full Board Review
Approval Date	6/21/2017
Expiration Date of the Study	4/25/2018
Funding Source(s)	1) BIOMET 3I, LLC<br>*Grant PI*: KRISTOFER JONES<br>*Grant Title*: A Multicenter, Double-Blind, Randomized, Saline-Controlled Study of a Single, Intra-Articular Injection of Autologous Protein Solution in Patients with Knee Osteoarthritis

Specific Conditions for Approval

-- **Research Participants Bill of Rights** - By California law, a copy of the Research Participants Bill of Rights in a language in which the participant is fluent must be given to all research participants in this study as there is a real or foreseeable risk of biomedical harm. Numerous translations are available for download on the HRPP website at http://www.ohrpp.research.ucla.edu/pages/bill-of-rights.

-- **Translations Needed** - Please submit translated copies of your consent documents as an amendment(s) before recruiting or consenting any subjects for whom these translations are required.

Documents Reviewed included, but were not limited to:

Document Name	Document Version #
16-001442 _ZimmerBiomet ICF CLEAN (09Jun2016).pdf.pdf	0.01

Important Note: Approval by the Institutional Review Board does not, in and of itself, constitute approval for the implementation of this research. Other UCLA clearances and approvals or other external agency or collaborating institutional approvals may be required before study activities are initiated. Research undertaken in conjunction with outside entities, such as drug or device companies, are typically contractual in nature and require an agreement between the University and the entity.

General Conditions of Approval
As indicated in the PI Assurances as part of the IRB requirements for approval, the PI has ultimate responsibility for the conduct of the study, the ethical performance of the project, the protection of the rights and welfare of human subjects, and strict adherence to any stipulations imposed by the IRB.

The PI and study team will comply with all UCLA policies and procedures, as well as with all applicable Federal, State, and local laws regarding the protection of human subjects in research, including, but not limited to, the following:

- Ensuring that the personnel performing the project are qualified, appropriately trained, and will adhere to the provisions of the approved protocol,
- Implementing no changes in the approved protocol or consent process or documents without prior IRB approval (except in an emergency, if necessary to safeguard the well-being of human subjects and then notifying the IRB as soon as possible afterwards),
- Obtaining the legally effective informed consent from human subjects of their legally responsible representative, and using only the currently approved consent process and stamped consent documents, as appropriate, with human subjects,
- Reporting serious or unexpected adverse events as well as protocol violations or other incidents related to the protocol to the IRB according to the OHRPP reporting requirements.
- Assuring that adequate resources to protect research participants (i.e., personnel, funding, time, equipment and space) are in place before implementing the research project, and that the research will stop if adequate resources become unavailable.
- Arranging for a co-investigator to assume direct responsibility of the study if the PI will be unavailable to direct this research personally, for example, when on sabbatical leave or vacation or other absences. Either this person is named as co-investigator in this application, or advising IRB via webIRB in advance of such arrangements.
