# Supplementary material for: Synovial fluid dual‐biomarker algorithm accurately differentiates osteoarthritis from inflammatory arthritis
Source: J Orthop Res. 2024 Dec 18;43(2):304–10. doi: 10.1002/jor.26005 (PMC11701394; doi:10.1002/jor.26005)
Supplement: Supplementary file 20 — Supporting information. [file JOR-43-304-s008.pdf]

WAKE FOREST
UNIVERSITY
HEALTH SCIENCES

Office of Research
INSTITUTIONAL REVIEW BOARD.

MEMORANDUM

To: Cristin Ferguson, M.D.
Surgery Orthopedics

From: Brian Moore, Director, IRB # 7
Institutional Review Board

Date: 10/6/2017

Subject: Human Protocol: IRB00045834
A Multicenter, Double-Blind, Randomized,Saline-Controlled Study of a Single, Intra-Articular Injection of Autologous Protein Solution in Patients with Knee Osteoarthritis

Study Documents:
Protocol Version: Protocol.pdf; Informed Consent Version: Wake Forest_V2 8.15.17_IFC_.revised 09.14.2017.clean copy_EEZ.docx; Investigator's Brochure: IDE 17069 Approval Letter 2016-07-29_For Distribution.pdf, PROGRESS IV_Investigator's Brochure_28JUN2016.pdf; Advertisements: 2016-08-26 PROGRESS IV-Patient Education Brochure.nationally prepared ad cannot be changed.pdf, Facebook Ad Campaign.nationally prepared ad cannot be changed.pdf, PROGRESS IV - FAQ - Patient FAQ - 9.12.16.nationally prepared ad cannot be changed.pdf, PROGRESS IV - IE Pocket Brochure - 8.8.16.nationally prepared ad cannot be changed.pdf, PROGRESS IV - Osteoarthritis Fact Sheet - 8.8.16.nationally prepared ad cannot be changed.pdf, PROGRESS IV - Social Media - Social Media Posts - 9.14.16.nationally prepared ad cannot be changed.docx, PROGRESS IV - Table Topper - 8.10.16.nationally prepared ad cannot be changed.pdf; Other Documents: PROGRESS IV - HCP FAQ - 8.8.16.pdf, PROGRESS IV - HCP Website Copy - 8.8.16.docx, PROGRESS IV - IE Pocket Brochure - 8.8.16.pdf, PROGRESS IV - Osteoarthritis Fact Sheet - 8.8.16.pdf, PROGRESS IV - PROGRESS IV Clinical Trial Fact Sheet - 8.8.16.pdf, PROGRESS IV - Trial Site Video Script - 8.8.16.pdf

The Institutional Review Board (IRB) has approved the above-named protocol and study documents, after review at a convened meeting on 9/6/2017. A submission requesting renewal together with a summary progress report must be submitted to the Board at least one month prior to 9/5/2018.

This approval includes a limited waiver of HIPAA authorization to identify potential subjects for recruitment into this research study, as allowed under 45 CFR 164.512. This temporary waiver provides access to protected health information (PHI) to confirm eligibility and facilitate initial contact, after which consent and HIPAA autorization will be sought. Access and use is limited to the minimum amount of PHI necessary to review eligibility criteria and to contact potential subjects.

This application indicates that advertising materials will be used for research purposes. Please consult with Creative Communications to ensure the appropriate visual identity is put forth.

Federal regulations and Board policy require that you promptly report to the Board for review/approval:

- Proposed changes in the research activity (e.g., protocol amendments; consent form revision; advertisements). Changes may not be initiated without IRB review and approval, unless necessary to eliminate an immediate hazard to subjects.

- Serious adverse events and unanticipated problems involving risks must be reported to the Board, institutional officials, FDA, sponsor and other regulatory agencies as required by the protocol, local policy and state or federal regulation.

Please provide a final report to the Board when the project is completed and Board approval can be terminated.

This IRB is in compliance with the requirements in Part 56, Subchapter D, Part 312 of the 21 Code of Federal Regulations published January 27, 1981 and Part 46, Subpart A of 45 CFR published January 26, 1981.

Medical Center Boulevard, Winston-Salem, NC 27157-1023 (336) 716-4542 / fax (336) 716-4480
