# Supplementary material for: Synovial fluid dual‐biomarker algorithm accurately differentiates osteoarthritis from inflammatory arthritis
Source: J Orthop Res. 2024 Dec 18;43(2):304–10. doi: 10.1002/jor.26005 (PMC11701394; doi:10.1002/jor.26005)
Supplement: Supplementary file 22 — Supporting information. [file JOR-43-304-s022.pdf]

DukeMedicine
Institutional Review Board for Clinical Investigations

NOTIFICATION OF IRB APPROVAL

**Protocol ID:**	Pro00085088
**Principal Investigator:**	Thorsten Seyler
**Protocol Title:**	A Multicenter, Double-Blind, Randomized, Saline-Controlled Study of a Single, Intra-Articular Injection of Autologous Protein Solution in Patients with Knee Osteoarthritis
**Sponsor/Funding Source(s):**	Zimmer Biomet
**Federal Funding Agency ID:**	

Date of Declared Concordance with federally funded grant, if applicable: N/A

The Duke University Health System Institutional Review Board for Clinical Investigations has conducted the following activity on the study cited above:

**Activity:**	Initial Review	**Review Type:** Full Committee Review
**Review Date:**	9/13/2017 IRB 03	
**Issue Date:**	10/12/2017	
**Expiration Date:**	9/13/2018	

DUHS IRB approval encompasses the following specific components of the study:

**Protocol, version/date:**	--V1.0 - 6/28/2016
**Summary, version/date:**	--9/27/2017
**Consent form reference date:**	--10/12/2017
**Investigator Brochure, version/date:**	--V1.0 - 6/28/2016
**Pediatric Risk Category:**	--

Other: --Waiver, Recruitment Material, Phone Script 10/2/2017, Patient Guide, Image Protocol Revision A, Work Sheets, nStride APS Kit Package Insert

The DUHS IRB has determined the specific components above to be in compliance with all applicable Health Insurance Portability and Accountability Act ("HIPAA") regulations.

This study expires at 12 AM on the Expiration Date cited above. At that time, all study activity must cease. If you wish to continue specific study activities directly related to subject safety, you must immediately email Jody Power at jody.power@duke.edu or call the IRB Office at 919-668-5111 and follow the instructions to reach the IRB Chair on call. Continuing review submissions (renewals) must be received by the DUHS IRB office 60 to 45 days prior to the Expiration Date.

No change to the protocol, consent form or other approved document may be implemented without first obtaining IRB approval for the change. Any proposed change must be submitted as an amendment. If necessary in a life-threatening situation, where time does not permit your prior consultation with the IRB, you may act contrary to the protocol if the action is in the best interest of the subject. You must notify the IRB of your action within five (5) working days of the event.

The Duke University Health System Institutional Review Board for Clinical Investigations (DUHS IRB), is duly constituted, fulfilling all requirements for diversity, and has written procedures for initial and continuing review of human research protocols. The DUHS IRB complies with all U.S. regulatory requirements related to the protection of human research participants. Specifically, the DUHS IRB complies with 45CFR46, 21CFR50, 21CFR56, 21CFR312, 21CFR812, and 45CFR164.508-514. In addition, the DUHS IRB complies with the Guidelines of the International Conference on Harmonization to the extent required by the U. S. Food and Drug Administration.

DUHS Institutional Review Board
2424 Erwin Rd | Suite 405 | Durham, NC | 919.668.5111
Federalwide Assurance No: FWA 00009025
