# Supplementary material for: Synovial fluid dual‐biomarker algorithm accurately differentiates osteoarthritis from inflammatory arthritis
Source: J Orthop Res. 2024 Dec 18;43(2):304–10. doi: 10.1002/jor.26005 (PMC11701394; doi:10.1002/jor.26005)
Supplement: Supplementary file 23 — Supporting information. [file JOR-43-304-s011.pdf]

Certificate of Action

**Investigator Name:** William M. Schnitz, MD	**Board Action Date:** 01/12/2018
**Investigator Address:** 3555 N.W. 58th Street, Suite 800<br>Oklahoma City, OK 73112, United States	**Approval Expires:** 09/30/2018<br>**Continuing Review Frequency:** Annually
**Sponsor:** Biomet Biologics, LLC<br>**Institution Tracking Number:**	**Sponsor Protocol Number:** APSS-44-00<br>**Amended Sponsor Protocol Number:**
**Study Number:** 1182348	**IRB Tracking Number:** 20161893
**Work Order Number:** 1-1056259-1	**Panel:** 2
**Protocol Title:** A Multicenter, Double-Blind, Randomized, Saline-Controlled Study of a Single, Intra-Articular Injection of Autologous Protein Solution in Patients with Knee Osteoarthritis	

THE FOLLOWING ITEMS ARE APPROVED:

This is to certify that the information contained herein is true and correct as reflected in the records of this IRB. WE CERTIFY THAT THIS IRB IS IN FULL COMPLIANCE WITH GOOD CLINICAL PRACTICES AS DEFINED UNDER THE U.S. FOOD AND DRUG ADMINISTRATION (FDA) REGULATIONS, U.S. DEPARTMENT OF HEALTH AND HUMAN SERVICES (HHS) REGULATIONS, AND THE INTERNATIONAL CONFERENCE ON HARMONISATION (ICH) GUIDELINES.

Investigator
Administrative Letter (09-21-2016) Pregnancy Test Requirement
Adverse Event Form #14999517.0 - As Submitted
Advertisement - Answering Message Script - Thank you for your interest #15143272.0 - As Submitted
Advertisement - Clinical Trials Fact Sheet - What Is A Clinical Trial #14858799.0 - As Submitted
Advertisement - Craigslist - If you have knee OA #16701056.0 - As Submitted
Advertisement - Facebook Advertising Campaign #15927431.0 - As Submitted
Advertisement - Flyer - Knee Osteoarthritis? You may Qualify #16507322.0 - As Submitted
Advertisement - Health Care Provider FAQ - Progress IV Clinical Trial FAQ #15562404.0 - As Submitted
Advertisement - Once osteoarthritis pain starts, it's (V.1 with logo placeholder) #17012718.0 - As Submitted
Advertisement - Once osteoarthritis pain starts, it's (V.1 without logo placeholder) #17012721.0 - As Submitted
Advertisement - Once osteoarthritis pain starts, it's (V.2 with logo placeholder) #17012720.0 - As Submitted
Advertisement - Once osteoarthritis pain starts, it's (V.2 without logo placeholder) #17012719.0 - As Submitted
Advertisement - Patient Education Brochure - Progress IV Clinical Trial Patient #14858797.1 - As Submitted
Advertisement - Patient FAQ - Progress IV Clinical Trial FAQ #14858803.1 - As Submitted
Advertisement - Poster - Knee Osteoarthritis? You may Qualify #16507323.0 - As Submitted
Advertisement - Pre-Screening Message Content - Thanks for your interest in #15143274.0 - As Submitted
Advertisement - Social Media Posts - Working in the yard a #14858795.1 - As Submitted
Advertisement - Table Topper - If You Have Knee OA #14858805.0 - As Submitted
Advertisement - Trial Site Video Recording - Osteoarthritis (OA) the most common type #14858793.1 - As Submitted
Advertisement - Website Copy (Patients) - OA of the knee is #14858794.1 - As Submitted
Clinical Trial Fact Sheet #14858804.0 - As Submitted
Clinical Trial FAQ for Health Care Providers #14858800.0 - As Modified
Demographics and Medication Use - Screening Visit #14999525.0 - As Submitted
Eligibility Criteria - Screening Visit #14999521.0 - As Submitted
EQ-5D Health Questionnaire - 2nd Injection Follow-Up Visit #14999572.0 - As Submitted
EQ-5D Health Questionnaire - Injection Visit #14999529.0 - As Submitted
EQ-5D Health Questionnaire - Month 1 Visit #14999535.0 - As Submitted
EQ-5D Health Questionnaire - Month 12 Visit #14999555.0 - As Submitted
EQ-5D Health Questionnaire - Month 3 Visit #14999540.0 - As Submitted
EQ-5D Health Questionnaire - Month 6 Visit #14999550.0 - As Submitted
EQ-5D Health Questionnaire #15174698.0 - As Submitted
Follow-Up Visit - 2nd Injection Follow-Up Visit #14999570.0 - As Submitted
Follow-Up Visit - Month 1 Visit #14999533.0 - As submitted
Follow-Up Visit - Month 12 Visit #14999552.0 - As Submitted
Follow-Up Visit - Month 3 Visit #14999537.0 - As Submitted
Follow-Up Visit - Month 6 Visit #14999543.0 - As Submitted
Health and Fitness 2017 June Article #16011845.0 - As Modified
Injection Visit #14999528.0 - As Submitted
Injection Visit 2 - Open-Label 2nd Injection Visit #14999557.0 - As Submitted
Monitoring Plan #14999499.0 - As Submitted
nStride Study Stipend Log #16529380.0 - As Submitted
Osteoarthritis Fact Sheet #14858802.0 - As Submitted
Pocket Brochure - Schedule of Visits and Inclusion-Exclusion Criteria #14858796.0 - As Submitted
Preparing for Procedure Day - Guide for Patients #16272081.0 - As Submitted
Pre-Screeners #16636954.0 - As Submitted
Protocol (06-28-2016) Version 1.0
Protocol Deviation Form #14999518.0 - As Submitted
Sample Processing - Injection Visit #14999532.0 - As Submitted
Statistical Analysis Plan #14999497.0 - As Submitted
Study Exit Form #14999520.0 - As Submitted
VAS Pain Assessment - 2nd Injection Follow-Up Visit #14999576.0 - As Submitted
VAS Pain Assessment - Injection Visit #14999531.0 - As Submitted
VAS Pain Assessment - Month 1 Visit #14999536.0 - As Submitted
VAS Pain Assessment - Month 12 Visit #14999556.0 - As Submitted
VAS Pain Assessment - Month 3 Visit #14999541.0 - As Submitted
VAS Pain Assessment - Month 6 Visit #14999551.0 - As Submitted
VAS Pain Assessment #15174696.1 - As Submitted
Website Copy - Health Care Providers #14858801.1 - As Submitted
WOMAC Osteoarthritis Index LK 3.1 - 2nd Injection Follow-Up Visit #14999571.0 - As Submitted
WOMAC Osteoarthritis Index LK 3.1 - Month 1 Visit #14999534.0 - As Submitted

WOMAC Osteoarthritis Index LK 3.1 - Month 12 Visit #14999554.0 - As Submitted
WOMAC Osteoarthritis Index LK 3.1 - Month 3 Visit #14999539.0 - As Submitted
WOMAC Osteoarthritis Index LK 3.1 - Month 6 Visit #14999548.0 - As Submitted
WOMAC Osteoarthritis Index LK 3.1 - Screening Visit #14999524.0 - As Submitted
WOMAC Osteoarthritis Index LK 3.1 #15174697.1 - As Submitted
Consent Form [S0]

Please note the following information:
The Board requires that all subjects must be able to consent for themselves to be enrolled in this study. This means that you cannot enroll incapable subjects who require enrollment by consent of a legally authorized representative.

NOTE: WIRB currently has NStride APS Kit Drug Brochure (06-28-2016, Version 1.0) on file.

THE IRB HAS APPROVED THE FOLLOWING LOCATIONS TO BE USED IN THE RESEARCH:
Lynn Health Science Institute, 3555 N. W. 58th Street, Suite 800, Oklahoma City, Oklahoma 73112

ALL IRB APPROVED INVESTIGATORS MUST COMPLY WITH THE FOLLOWING:
As a requirement of IRB approval, the investigators conducting this research will:

- Comply with all requirements and determinations of the IRB.
- Protect the rights, safety, and welfare of subjects involved in the research.
- Personally conduct or supervise the research.
- Conduct the research in accordance with the relevant current protocol approved by the IRB.
- Ensure that there are adequate resources to carry out the research safely.
- Ensure that research staff are qualified to perform procedures and duties assigned to them during the research.
- Submit proposed modifications to the IRB prior to their implementation.
    - Not make modifications to the research without prior IRB review and approval unless necessary to eliminate apparent immediate hazards to subjects.
- Submit continuing review reports when requested by the IRB.
- Submit a closure form to close research (end the IRB’s oversight) when:
    - The protocol is permanently closed to enrollment
    - All subjects have completed all protocol related interventions and interactions
    - For research subject to federal oversight other than FDA:
- No additional identifiable private information about the subjects is being obtained
- Analysis of private identifiable information is completed
- If research approval expires, stop all research activities and immediately contact the IRB.
- Promptly report to the IRB the information items listed in the IRB's "Prompt Reporting Requirements” available on the IRB's Web site.
- Not accept or provide payments to professionals in exchange for referrals of potential subjects (“finder’s fees.”)
- Not accept payments designed to accelerate recruitment that are tied to the rate or timing of enrollment (“bonus payments”) without prior IRB approval.
- When required by the IRB ensure that consent, permission, and assent are obtained and documented in accordance with the relevant current protocol as approved by the IRB.
- Promptly notify the IRB of any change to information provided on your initial submission form.

Consistent with AAHRPP’s requirements in connection with its accreditation of IRBs, the individual and/or organization shall promptly communicate or provide, the following information relevant to the protection of human subjects to the IRB in a timely manner:

- Upon request of the IRB, a copy of the written plan between sponsor or CRO and site that addresses whether expenses for medical care incurred by human subject research subjects who experience research related injury will be reimbursed, and if so, who is responsible in order to determine consistency with the language in the consent document.
- Any site monitoring report that directly and materially affects subject safety or their willingness to continue participation. Such reports will be provided to the IRB within 5 days.
- Reports from any data monitoring committee, data and safety monitoring board, or data and safety monitoring committee in accordance with the time frame specified in the research protocol.
- Any findings from a closed research when those findings materially affect the safety and medical care of past subjects. Findings will be reported for 2 years after the closure of the research.

If your research site is a HIPAA covered entity, the HIPAA Privacy Rule requires you to obtain written authorization from each research subject for any use or disclosure of protected health information for research. If your IRB-approved consent form does not include such HIPAA authorization language, the HIPAA Privacy Rule requires you to have each research subject sign a separate authorization agreement. ”

Federal regulations require that the IRB conduct continuing review of approved research. You will receive Continuing Review Report forms from this IRB when the expiration date is approaching.

Thank you for using this WCG IRB to provide oversight for your research project.

DISTRIBUTION OF COPIES:

Contact, Company

Kathi Shaw, Lynn Health Science Institute
Meghan Kulaszewski, IMARC Research Inc.
William M. Schnitz, MD, Lynn Health Science Institute
Krista Toler, Zimmer Biomet
WIRB Translations Dept., WIRB USA
Ann Blanton, Biomet Biologics
